# Largest pediatric scrotal lipoma with perineal extension: a rare case expanding the anatomic spectrum and diagnostic pitfalls

**DOI:** 10.1186/s12894-026-02240-z

**Published:** 2026-07-21

**Authors:** Ahmed Abdelmohsen

**Affiliations:** Department of Pediatric Surgery, Faculty of Medicine, Al-Azhar University, Cairo, Egypt

**Keywords:** Scrotal lipoma, Giant lipoma, Perineal extension, Scrotal mass, Scrotal swelling, Pediatric surgery, Case report

## Abstract

**Background:**

Scrotal lipomas are exceptionally rare in children, with only a few cases reported worldwide and most measuring less than 7 cm. Giant pediatric scrotal lipomas with multi-compartmental perineal extension have not been previously described. This case presents the largest documented pediatric scrotal lipoma, with an analytic framework for understanding diagnostic pitfalls and rare visual documentation of surgical anatomy.

**Case Presentation:**

A 5-year-old boy presented with six months of progressive left scrotal swelling clinically consistent with a large hydrocele. Ultrasound suggested heterogeneous fatty tissue, while MRI performed elsewhere was interpreted as omental herniation. Standard inguinal exploration for presumed hydrocele revealed a normal testis without a hernia or fluid collection, prompting intraoperative diagnostic reassessment. Subsequent scrotal exploration identified a well-encapsulated lipoma measuring 14 × 12 × 7 cm, located between the dartos and scrotal skin with deep extensions to the pubic arch, penile root, and pelvic floor. Complete excision was achieved while preserving all neurovascular structures. Histopathology confirmed mature lipoma. At one-year follow-up, the patient remained asymptomatic with symmetric testes and no recurrence.

**Conclusions:**

This case demonstrates how ultra-rare pediatric conditions can closely mimic common scrotal diseases, leading to diagnostic anchoring despite supportive imaging. It documents, to the best of our knowledge, the largest pediatric scrotal lipoma to date and the only case with extensive perineal extension. Intraoperative diagnostic flexibility, combined with meticulous anatomical dissection, enables safe and complete excision with excellent functional outcomes.

**Supplementary Information:**

The online version contains supplementary material available at 10.1186/s12894-026-02240-z.

## Background

 Lipomas are common benign soft tissue tumors in adults, with a prevalence of 2.1 per 1,000 people [1]. However, scrotal involvement is exceptionally rare, particularly in the pediatric population. Giant lipomas exceeding 10 cm represent only 1% of all lipomas [2]. Among documented pediatric scrotal lipomas, the largest previously reported measured 6.5 × 4.5 × 2 cm in a 9-year-old boy [3]. Most pediatric cases occur in neonates or infants, often associated with congenital anomalies [4–6].

Primary scrotal lipomas can be classified by anatomical origin: paratesticular (arising from spermatic cord, epididymis, or tunica vaginalis) or extratesticular (from preperitoneal fat, perineal fascia, or scrotal wall) [7, 8]. Accurate preoperative diagnosis remains challenging due to clinical similarity to more common conditions such as hydrocele or inguinal hernia [2, 9]. This diagnostic uncertainty can lead to unexpected intraoperative findings requiring adaptive surgical management [10].

Given their rarity, the natural history, anatomical patterns of extension, and optimal surgical management of pediatric scrotal lipomas remain incompletely characterized in the published literature. Notably, multi-compartmental perineal extension has not been previously reported in a child, and the mechanisms leading to diagnostic misinterpretation in such cases have not been analyzed systematically.

Here, we present a 14 × 12 × 7 cm primary scrotal lipoma in a 5-year-old boy—the largest pediatric case documented to date and the only one demonstrating extensive extension into the pubic arch, penile root, and pelvic floor compartments. Beyond detailing the clinical, radiologic, and operative findings, this case offers systematic analysis of the diagnostic pitfalls, cognitive biases, and intraoperative decision-making strategies encountered when preoperative assumptions fail.

This report therefore contributes both a unique clinical case and broader insights relevant to surgeons, radiologists, and clinicians managing pediatric scrotal masses. This case is further strengthened by high-quality photographs that clearly illustrate the unusual anatomical extensions of the lipoma, offering high-fidelity operative documentation that is absent from prior reports of this condition.

## Case presentation

This case report was prepared in accordance with the CARE (CAse REport) guidelines [11]. A 5-year-old previously healthy boy presented with progressive left scrotal swelling over 6 months. His parents reported steady size increase causing marked asymmetry and mild ambulation difficulty. The child was asymptomatic with no pain, fever, or urinary symptoms. There was no history of trauma, undescended testis, or family history of soft tissue tumors.

**Fig. 1:**
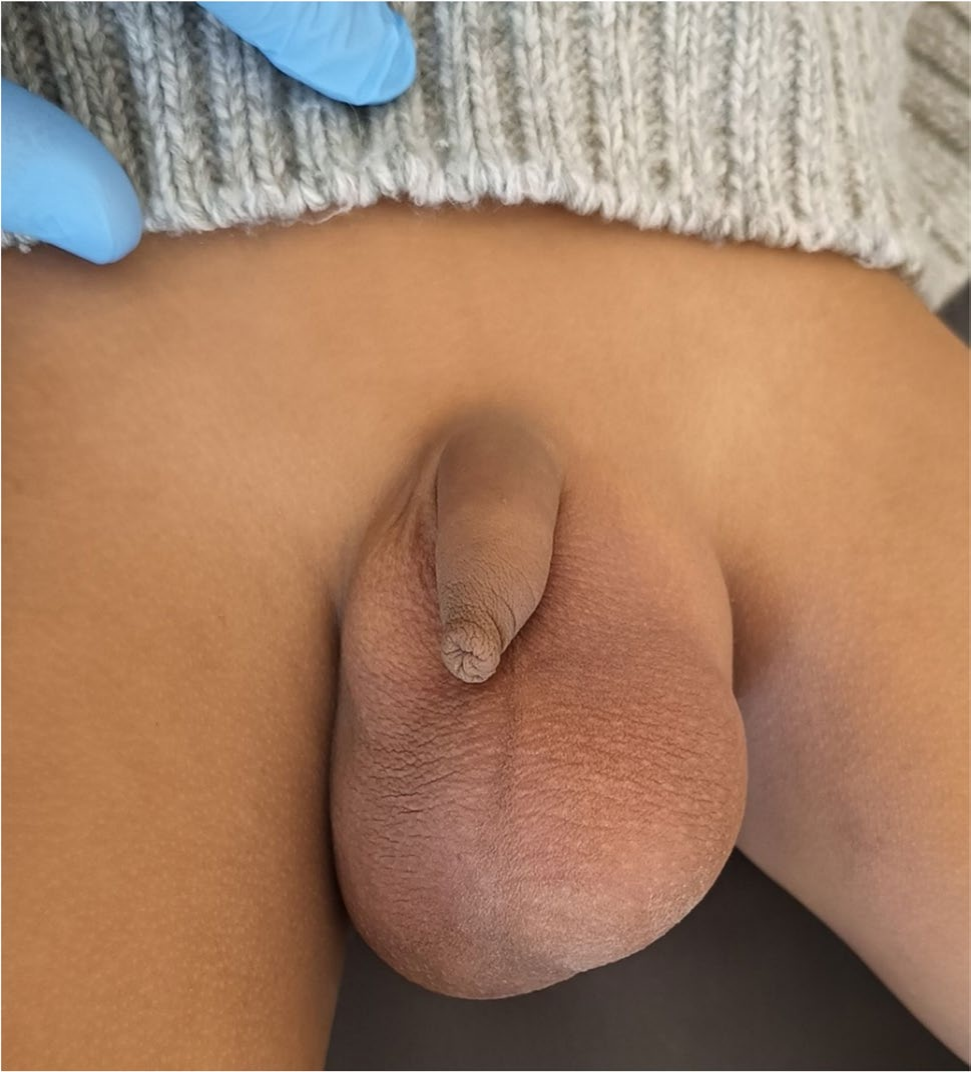
Preoperative appearance demonstrating marked left scrotal asymmetry with massive enlargement mimicking the typical clinical presentation of large hydrocele in a pediatric patient. Note the smooth contour and absence of erythema or other signs of acute pathology

Physical examination revealed marked scrotal asymmetry with significant left hemiscrotum enlargement (Fig. 1). A large (approximately 11 cm), soft, non-tender, not reducible swelling occupied the entire left hemiscrotum extending into the inguinal region, with pseudofluctuation leading to initial clinical impression of large hydrocele rather than inguinal hernia. The left testis was difficult to palpate due to mass size; the right testis was normal.

## Laboratory investigations

Routine preoperative tests—including complete blood count, renal and liver function tests, and urinalysis—were normal. Tumor markers were not obtained because the clinical impression favored benign hydrocele. 

## Imaging

Scrotal ultrasonography demonstrated a large heterogeneous mass within the left hemiscrotum containing internal septations and areas of increased echogenicity. The left testis appeared compressed superiorly but maintained normal vascular flow. The radiologic impression favored herniated omentum within a tense inguinoscrotal hernia.

An MRI performed at an outside facility revealed a well-circumscribed 12 × 11 cm mass with high T1/T2 signal intensity consistent with adipose tissue. Despite these fat-signal characteristics, the lesion was interpreted as “omental herniation through an occult inguinal hernia.” The patient was referred to our institution with the radiology report and a primary clinical diagnosis of large hydrocele; the original cross-sectional images were not available for independent review at the time of planned surgery.

## Surgical procedure

**Fig. 2:**
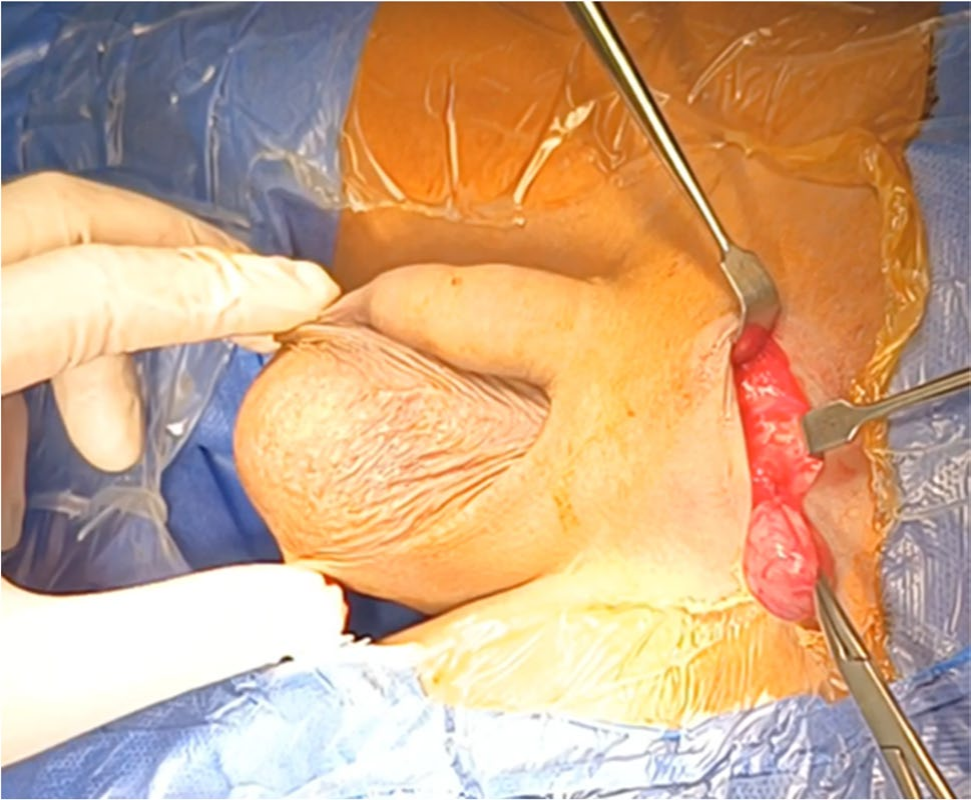
Intraoperative view through inguinal incision showing normal left testis with intact tunica vaginalis and normal gubernacular attachment to the scrotum. No hernial sac, fluid collection, or intrascrotal mass was identified during inguinal exploration, prompting diagnostic reassessment

Under general anesthesia, initial inguinal exploration was performed. No hernial sac was identified at the internal ring or within the inguinal canal. Following the spermatic cord distally revealed a normal left testis with an unexpectedly empty scrotal compartment. (Fig. 2). Intraoperative exploration of the internal inguinal ring confirmed no patent processus vaginalis bilaterally, effectively excluding communicating hydrocele and indirect inguinal hernia as the underlying diagnosis. This unexpected finding prompted intraoperative diagnostic reassessment.

**Fig. 3:**
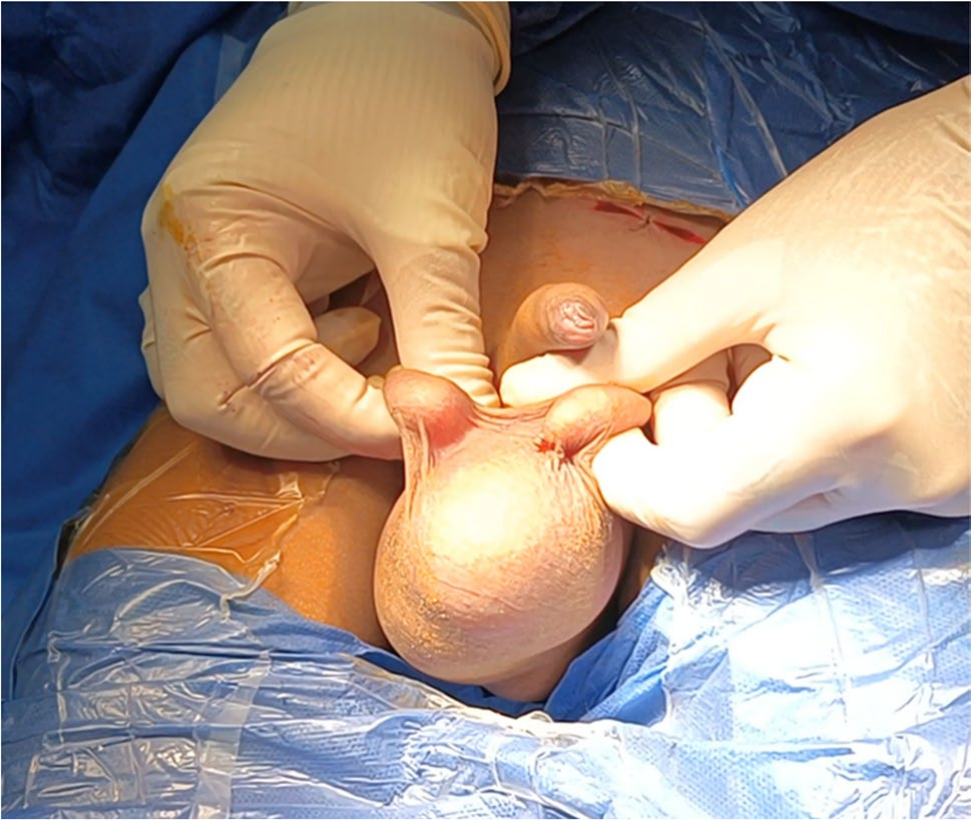
Intraoperative examination demonstrating testicular separation from the underlying mass. Bilateral gentle pinching of the scrotal skin from above clearly shows the testis as a distinct, mobile structure separate from the underlying pathology, confirming extratesticular location of the mass

Given the discrepancy between clinical expectations and intraoperative findings, a structured intraoperative reassessment was undertaken. A bilateral testicular separation maneuver—gently pinching the scrotal skin above each testis—confirmed that the testes were distinct, mobile structures separate from the underlying mass (Fig. 3). These findings indicated an extratesticular lesion located outside the tunica vaginalis.

It became clear that the pathology represented a primary scrotal lipoma arising from the fat lobules of the dartos tunica, situated within the subcutaneous scrotal wall between the dartos layer and the overlying scrotal skin—corresponding to Fujimura’s Type 3 primary scrotal lipoma, the rarest anatomical variant of primary scrotal lipoma [8].

A 10 cm longitudinal median raphe scrotal incision was made. The scrotal skin and dartos were carefully dissected from a large yellowish, multilobulated fatty mass with intact fibrous capsule. The main tumor bulk was gradually mobilized and it was much bigger than previously expected from the examination.

**Fig. 4:**
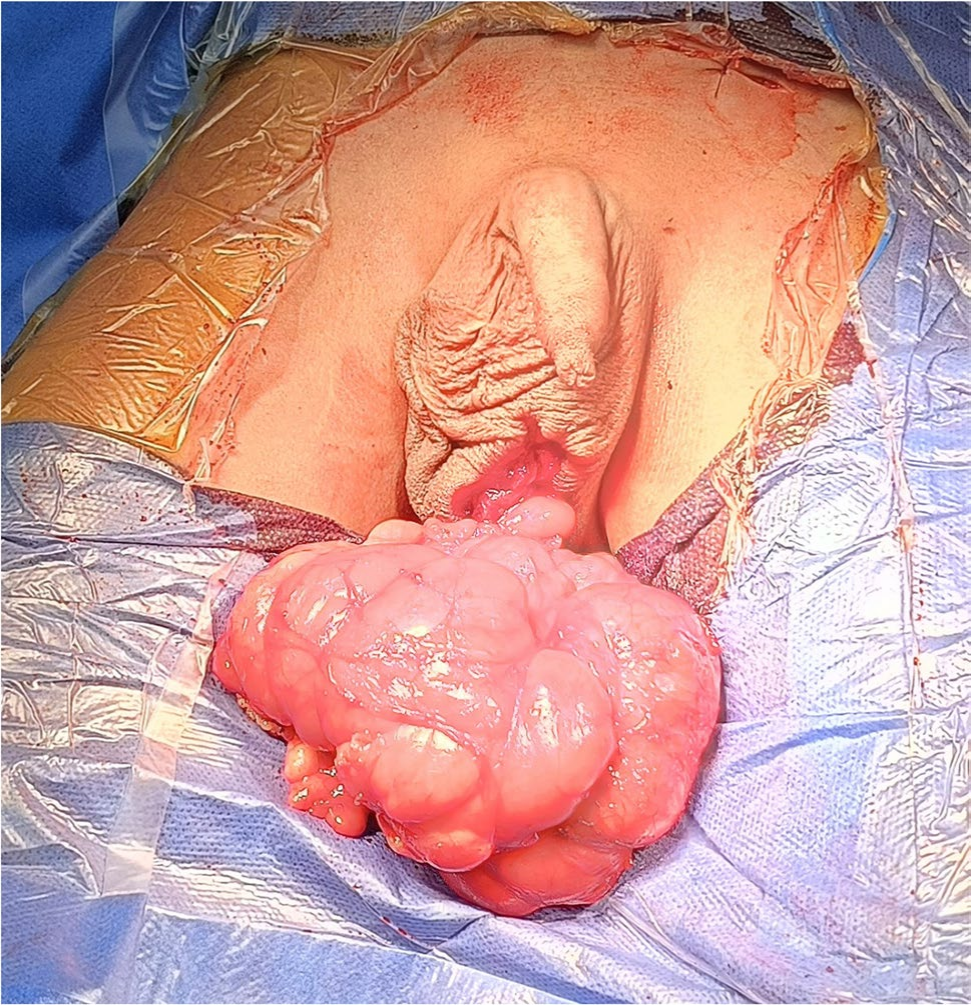
Extraction of the main bulk of the lipoma through the longitudinal scrotal incision. Note the well-encapsulated nature of the mass and its yellow, lobulated appearance characteristic of mature lipomatous tissue

**Fig. 5:**
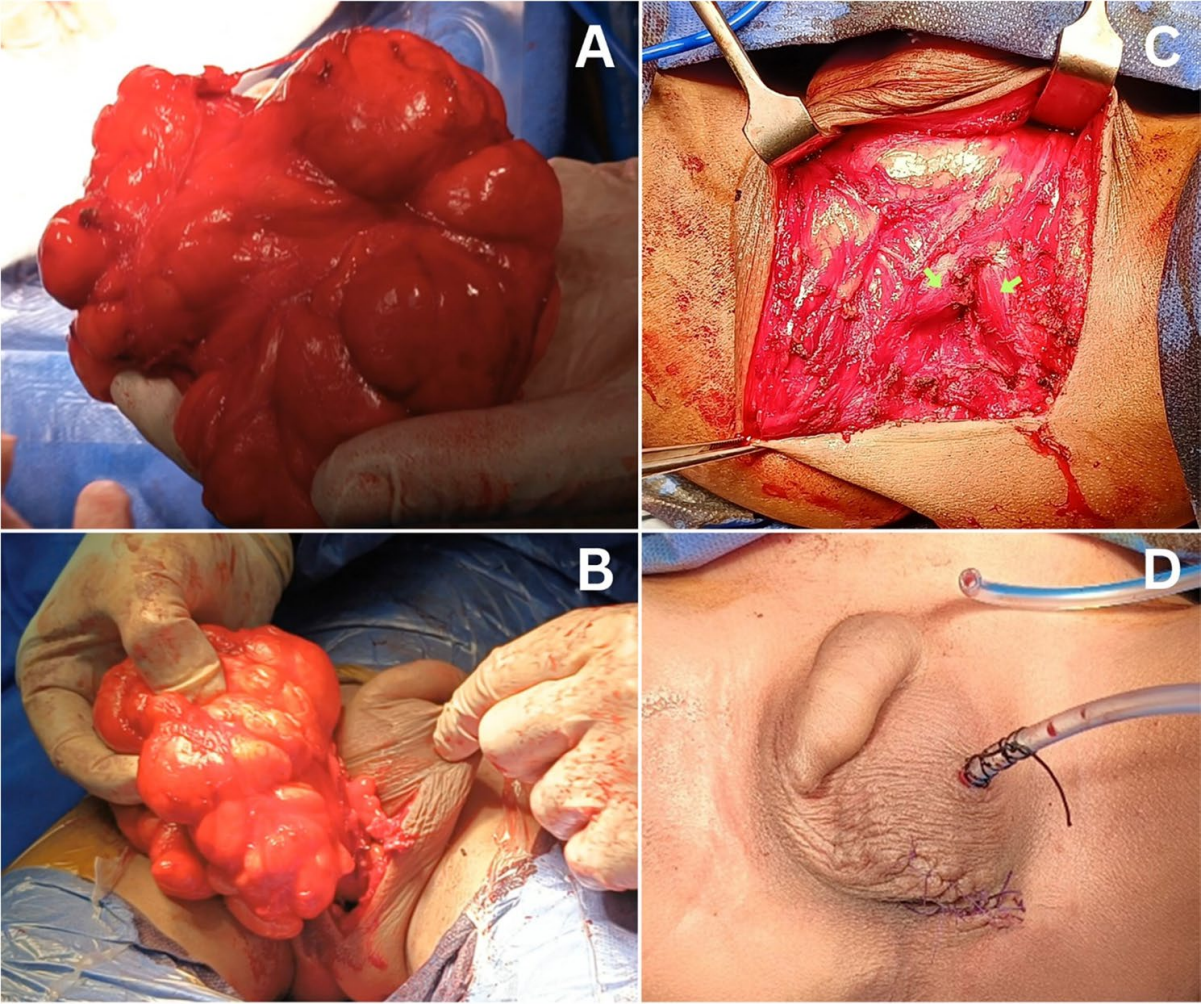
Deep perineal extension of the lipoma. **A** Post-excision view of the perineal surface of the lipoma. **B** Extensive involvement of the perineum requiring extension of the scrotal incision. **C** Full perineal exploration through the redundant scrotum demonstrating complete excision from the involved structures, including attachments to the pubic bones and root of the penis (green arrows) and pelvic floor musculature. **D** Immediate postoperative appearance showing marked reduction in scrotal size to nearly normal dimensions following complete excision. A suction drain is visible, placed to prevent hematoma formation. Note the symmetric appearance of both hemiscrotum compared to the preoperative massive asymmetry

Complete mobilization was impeded by extensive perineal attachments. The scrotal incision was extended with a small perineal extension (Fig. 4), revealing tumor attachments to:

1. Inferior pubic arch and left pubic ramus.
2. Root of penis and crural fascia (green arrows, Fig. 5).
3. Superficial perineal pouch and perineal body.

These attachments were systematically divided using sharp dissection and cautery, with extreme care to avoid urethral, penile neurovascular and anal sphincter injury. After careful dissection, the tumor was completely excised en bloc. A suction drain was placed. The scrotum immediately shrank to nearly normal dimensions (Fig. 5).

Operative time was 70 min with an estimated blood loss of 20 mL. No intraoperative complications occurred, and no transfusion was required.

## Histopathology and follow-up

Gross pathology revealed a well-encapsulated lobulated fatty tumor measuring 14 × 12 × 7 cm and weighing 420 g. Microscopy demonstrated mature adipocytes with thin fibrovascular septae and no lipoblasts, atypia, or mitotic activity, confirming benign mature lipoma. The final diagnosis was benign mature lipoma with complete excision and negative margins.

The patient had an uneventful recovery. The drain was removed on postoperative day 2, and he was discharged on day 3. Follow-up at 2 weeks, 6 weeks, 3 months, 6 months, and 1 year showed excellent wound healing, symmetric scrotal appearance, and normal testicular growth on ultrasonography with no evidence of recurrence.

The patient’s parents had been increasingly distressed by the progressive and visible enlargement over 6 months, prompting them to seek a surgeon experienced in managing complex pediatric cases given the size of the mass. Despite their anxiety, the child remained in overall good general condition throughout, with no systemic compromise. Following complete excision and restoration of near-normal scrotal dimensions, the family expressed considerable relief and satisfaction with both the functional and cosmetic outcome at the 12-month follow-up visit. Table 1 summarizes the full clinical timeline.

**Table 1:** Clinical timeline

Time Point	Event	Clinical Details	References
6 months before presentation	Onset of symptoms	Progressive left scrotal swelling noticed by parents	-
Initial presentation	Clinical examination	Marked left scrotal asymmetry; soft, non-tender, non-reducible mass (~ 11 cm); pseudofluctuation present	Figure 1
Week 1	Scrotal ultrasonography	Large heterogeneous mass with internal septations; left testis compressed but normal vascularity; impression: herniated omentum	-
Week 2	MRI (outside facility)	Well-circumscribed 12 × 11 cm mass with high T1/T2 signal (fat characteristics); interpreted as omental herniation	-
Week 3	Surgical intervention	Inguinal exploration: no hernia, normal testis; scrotal exploration: complete excision of 14 × 12 × 7 cm lipoma with perineal extension	Figs. 2-5
Postoperative Day 2	Drain removal	Uneventful recovery; drain removed	-
Postoperative Day 3	Hospital discharge	Patient discharged home in good condition	-
2 weeks - 6 months postoperative	Follow-up	Excellent wound healing; no complications; symmetric scrotal appearance; normal testicular examination; Ultrasonography: normal testicular growth bilaterally	-
12 months postoperative	Final follow-up	No recurrence; symmetric scrotum; normal testicular development	-

*MRI *Magnetic Resonance Imaging

## Discussion and conclusions

Scrotal lipomatous tumors represent rare benign entities in pediatric populations, with primary scrotal lipomas being exceedingly uncommon. The classification of intrascrotal lipomas has evolved to reflect their anatomical origin and relationship to scrotal structures. Leyson et al. [7] divided intrascrotal lipomas into two broad categories: paratesticular and extratesticular, with multiple subdivisions according to their site of origin. Fujimura [8] later described a more practical classification into three distinct types: (1) scrotal lipomas originating from subcutaneous fatty tissue posterior to the spermatic cord that spread into the scrotum; (2) spermatic cord and tunica vaginalis lipomas arising from fat tissue within or outside the spermatic cord that develop within the cord; and (3) primary scrotal lipomas originating from fat lobules within the dartos tunica of the scrotum—the rarest form [8, 12].

## Rarity in the pediatric population

Large scrotal lipomas in children are exceedingly rare. Kong et al. [3] reported a 5-year-old boy with a 6.0 × 4.0 × 3.0 cm right scrotal lipoma—the only comparable recent pediatric case. Hussein et al. [12] conducted a systematic review identifying 18 primary scrotal lipomas with mean patient age of 40 years; only two were pediatric cases: a 2-year-old with a 6.3 × 5.2 × 5.7 cm lipoma (described by Gemilang et al. [13] as the largest giant pediatric scrotal lipoma at that time) and a 1-month-old neonate with a 3 × 1.5 cm lipoma reported by Kim et al. [4].

Most pediatric cases occur in neonates or infants, often associated with congenital anomalies. Kim et al. [4] described a primary scrotal lipoma presenting as a pendulous midline mass in a newborn. Kavecan et al. [5] reported a peduncular perineal lipoma attached to an accessory scrotum in a newborn. Park et al. [6] reviewed perineal lipomas associated with scrotal anomalies, including accessory scrotum and penoscrotal transposition, but these cases involved congenital malformations rather than isolated massive scrotal lipomas in anatomically normal patients.

Our case at 14 × 12 × 7 cm represents, to the best of our knowledge, the largest pediatric scrotal lipoma documented worldwide and the only one with extensive multi-compartmental perineal involvement in a child with normal scrotal anatomy (Table 2).

**Table 2:** Comparative analysis of pediatric scrotal lipomas

Author	Age	Size (cm)	Extension	Preoperative Diagnosis	Surgical Approach
Kim 2009 [4]	1 month	3 × 1.5	Scrotal wall	Not stated	Scrotal
Gemilang 2023 [13]	2 years	6.3 × 5.2 × 5.7	Scrotal wall	Lipoma	Scrotal
Kong 2024 [3]	5 years	6.0 × 4.0 × 3.0	Right scrotum	Lipoma on ultrasound	Scrotal
Hussein 2023 [12]	9 years	6.5 × 4.5 × 2	Left scrotum	Lipoma on MRI	Combined inguinal and scrotal
Present case	5 years	14 × 12 × 7	Left scrotum + multi-compartmental perineum (pubic arch, penile root, pelvic floor)	Hydrocele/hernia	Inguinal exploration (negative), then scrotal

## Clinical reasoning and diagnostic challenges 

Reasoning and Diagnostic ChallengesIn pediatric practice, scrotal swelling in a 5-year-old most commonly represents hydrocele or inguinal hernia. Hydrocele remains primarily a clinical diagnosis in children, with imaging serving a supportive rather than definitive role. The soft, compressible nature of lipomas creates pseudo-fluctuation that closely mimics hydrocele on physical examination. Kong et al. [3] emphasized that transillumination—while traditional—is not reliable in children, as thin bowel and fatty tissue can transmit light, creating false-positive results.

Diagnostic challenges arise from unfamiliarity with these rare entities, ultrasound operator dependence and technical limitations, and cognitive biases favoring common diagnoses such as hydrocele or inguinal hernia [14, 15]. Misdiagnosis rates are substantial: Hussein et al. [12] documented only 44% accurate preoperative diagnosis rate in adult scrotal lipomas and 25% in pediatric cases. The critical distinction between lipoma and lipoblastoma in children carries significant clinical importance, as lipoblastoma demonstrates different epidemiology (predominantly < 3 years, male 3:1), characteristic imaging features (lobulation, T1 mosaic pattern on MRI), and higher recurrence rates (14–25%) requiring minimum 5-year follow-up [16–18].

The diagnostic pathway in our case—from clinical impression through appropriate surgical approach to intraoperative adaptation—reflects sound clinical practice rather than diagnostic error. The inguinal approach is the standard of care for pediatric hydrocele, allowing for high ligation of the processus vaginalis and direct inspection of the internal ring to exclude concomitant hernia. The extreme rarity of pediatric scrotal lipomas (fewer than 30 documented cases worldwide across all ages [12]) makes preoperative diagnosis particularly challenging, even with characteristic imaging findings.

Yada et al. [10] reported that among 11 cases of intrascrotal lipoblastoma—a related benign fatty tumor—accurate preoperative diagnosis was achieved in only one case. This diagnostic uncertainty often necessitates surgical exploration. In the adult literature, Sim and Park [9] reported that lipomas of the spermatic cord frequently present clinically as inguinoscrotal hernias, with diagnosis confirmed only at surgery.

The cognitive bias toward common diagnoses is well-established in clinical reasoning. When faced with a soft scrotal mass in a young child, the estimated pretest probability of hydrocele approaches 60–70% [19, 20], while the pretest probability of scrotal lipoma is essentially zero given the documented worldwide rarity. Even pathognomonic MRI findings demonstrating fat signal were reinterpreted within this clinical context to favor the more common diagnosis of herniated omentum.

When surgical findings diverge from preoperative expectations, systematic intraoperative examination becomes critical. The examination technique employed in this case—bilateral pinching of scrotal skin to demonstrate testicular separation from the underlying mass—proved valuable for confirming the extratesticular nature and guiding the subsequent surgical approach. While this maneuver may be intuitively performed by experienced surgeons, explicit description may benefit others encountering similar unexpected findings.

The extensive perineal involvement in our case is unprecedented in pediatric scrotal lipomas. Previous reported cases involved isolated scrotal wall lipomas without significant perineal extension [3, 4, 12, 13]. The multi-compartmental attachments to pubic arch, penile root, crural fascia, and pelvic floor structures required meticulous anatomical dissection to avoid injury to critical structures including the urethra, penile neurovascular bundle, and anal sphincter complex.

Differentiating lipoma from lipoblastoma is essential in pediatric patients due to differences in prognosis and recurrence. Lipoblastoma typically affects children younger than 3 years and displays lobulated architecture with immature adipocytes, with recurrence rates up to 25% [16–18]. Histopathology in this case demonstrated mature adipocytes without lipoblasts or atypia, confirming benign lipoma, consistent with the patient’s older age and clinical course.

## Lessons learned and clinical implications

This case provides several practical insights:

1. Rare lesions may present exactly like common conditions, necessitating reconsideration when clinical and imaging findings diverge.
2. Intraoperative diagnostic flexibility should be considered an essential component of managing pediatric scrotal masses.
3. Complex perineal extensions are not contraindications to complete excision, provided dissection is meticulous and anatomically oriented.
4. Diagnostic anchoring and cognitive bias may explain misinterpretation of even classic MRI features, highlighting the need for cautious interpretation when imaging does not fully align with operative findings.

An additional merit of this report is the inclusion of clear, high-quality preoperative and intraoperative photographs that demonstrate the anatomical relationships of the mass, including its deep perineal extensions. Given the extreme rarity of pediatric scrotal lipomas and the absence of comparable visual documentation in the literature, these images provide valuable educational insight into the surgical anatomy and operative strategy required for safe excision. Acknowledged limitations of this report include the absence of independent preoperative MRI image review, as the patient was referred with a radiology report only and original cross-sectional images were unavailable at our institution. Additionally, intraoperative ultrasonography was not utilized, as it is not routinely available in public pediatric surgical centers in low-to-middle-income settings and was not indicated for the primary operating diagnosis of hydrocele. These constraints reflect real-world practice in resource-limited environments and, importantly, the manual testicular separation maneuver provided equivalent anatomical localisation to guide surgical decision-making.

In conclusion, this case expands the clinical and anatomical spectrum of pediatric scrotal lipomas, representing to the best of our knowledge, the largest and most deeply invasive lesion described to date. It highlights the limitations of clinical judgment and imaging in rare conditions and demonstrates that complete excision with preservation of function is achievable through careful surgical planning. The lessons derived from this case are relevant for improving diagnostic accuracy and intraoperative decision-making in pediatric scrotal and perineal masses.

## Supplementary Information

Supplementary Material 1.

## Data Availability

All data generated or analyzed during this case report are included in this published article. Original clinical records, imaging studies, and pathology reports are retained in the patient’s medical record and can be made available upon reasonable request to the corresponding author, subject to patient privacy regulations.

## Abbreviations

CARE: CAse REport guidelines
MRI: Magnetic Resonance Imaging
T1: T1-weighted MRI sequence
T2: T2-weighted MRI sequence
