# Supplementary material for: Largest pediatric scrotal lipoma with perineal extension: a rare case expanding the anatomic spectrum and diagnostic pitfalls
Source: BMC Urol. 2026 Jul 21;26:174. doi: 10.1186/s12894-026-02240-z (PMC13390228; doi:10.1186/s12894-026-02240-z)
Supplement: Supplementary file 1 — Supplementary Material 1. [file 12894_2026_2240_MOESM1_ESM.docx]

**CARE Case Report Checklist**

| ☑ | ☐ | ☑ = Present and addressed in manuscript ☐ = Not applicable / not required for this case |
| --- | --- | --- |

| **#** | **Item** | **CARE Element** | **Where Addressed in Manuscript** | **Checklist Status** |
| --- | --- | --- | --- | --- |
| **1** | Title | Identifies the article as a case report | Title: 'Largest Pediatric Scrotal Lipoma with Perineal Extension: Expanding the Anatomic Spectrum and Diagnostic Pitfalls of a Rare Entity' | **☑ Present** |
| **2** | Keywords | 2–5 key words identifying diagnoses or interventions, including 'case report' | Keywords: Scrotal lipoma; Giant lipoma; Perineal extension; Scrotal mass; Pediatric surgery; Case report | **☑ Present** |
| **3a** | Abstract — Background | What is unique about this case and what does it add to the medical literature? | Abstract Background: describes rarity, absence of prior multi-compartmental perineal extension in children, and largest documented size | **☑ Present** |
| **3b** | Abstract — Case Presentation | Chief complaint, diagnosis, interventions, outcomes | Abstract Case Presentation: age, symptom duration, imaging findings, operative approach, lipoma dimensions, histopathology, 1-year follow-up | **☑ Present** |
| **3c** | Abstract — Conclusions | What are the main 'take-away' lessons from this case? | Abstract Conclusions: diagnostic anchoring, largest lipoma documented to our knowledge, intraoperative flexibility, functional outcomes | **☑ Present** |
| **4** | Background | Briefly summarises why this case is unique; background context and literature; why it merits reporting | Background section: epidemiology of scrotal lipomas, classification (Leyson [7], Fujimura [8]), diagnostic challenges [2,9], gaps in literature, rationale for reporting | **☑ Present** |
| **5a** | Patient Info — De-identified | Age, sex, ethnicity, occupation; chief complaints; relevant medical, family, and psychosocial history | Case Presentation: 5-year-old previously healthy boy; progressive scrotal swelling 6 months; no trauma, undescended testis, or family history of soft tissue tumours. Patient de-identified; no occupation relevant at this age. | **☑ Present** |
| **5b** | Medical / Surgical History | Relevant past interventions and their outcomes | No prior surgeries or interventions; no relevant past medical history stated | **☑ Present** |
| **6** | Clinical Findings | Relevant physical examination findings | Physical Examination: marked scrotal asymmetry, ~11 cm soft non-tender non-reducible mass, pseudofluctuation, left testis impalpable, right testis normal (Figure 1) | **☑ Present** |
| **7** | Timeline | Chronological summary of key diagnostic and therapeutic events | Table 1 (Clinical Timeline): symptom onset through 12-month follow-up, all diagnostic steps and surgical milestones listed chronologically | **☑ Present** |
| **8a** | Diagnostic Assessment — Methods | Diagnostic testing including laboratory tests and imaging | Laboratory Investigations: full blood count, renal/liver function tests, urinalysis — normal. Imaging: scrotal US (heterogeneous mass, septations, compressed testis); MRI 12×11 cm T1/T2 hyperintense mass (outside facility, report only) | **☑ Present** |
| **8b** | Diagnostic Assessment — Differentials | Diagnostic reasoning including other diagnoses considered | Clinical Reasoning section: hydrocele excluded (intraoperative — no fluid, no patent processus vaginalis bilaterally); inguinal hernia excluded (no hernial sac at internal ring); lipoblastoma differentiated by histopathology (mature adipocytes, no lipoblasts) | **☑ Present** |
| **8c** | Diagnostic Assessment — Prognosis | Prognostic characteristics when applicable | Histopathology: mature adipocytes, no lipoblasts, no atypia, no mitotic activity — confirmed benign mature lipoma; negative margins. No recurrence at 12 months. | **☑ Present** |
| **9** | Therapeutic Intervention | Types of interventions (e.g., pharmacological, surgical); administration and dosing; changes over time; rationale | Surgical Procedure: inguinal exploration under GA → scrotal + perineal exploration → en bloc excision 14×12×7 cm lipoma; 70 min operative time; 20 mL blood loss; suction drain placed. Attachments to pubic arch, penile root, crural fascia, perineal body divided with sharp dissection/cautery. | **☑ Present** |
| **10** | Follow-up & Outcomes | Clinician- and patient-assessed outcomes; important follow-up results; intervention adherence; adverse and unanticipated events | Histopathology and Follow-up section: drain removed POD 2; discharge POD 3; follow-up at 2 wks, 6 wks, 3, 6, 12 months — excellent wound healing, symmetric scrotal appearance, normal testicular growth on US, no recurrence. | **☑ Present** |
| **11** | Patient Perspective | Patient's (or family's) perspective of the care received | End of Histopathology and Follow-up section: parents increasingly distressed by progressive visible enlargement; sought experienced surgeon given mass size; child in overall good general condition throughout; family expressed considerable relief and satisfaction at 12-month follow-up. | **☑ Present** |
| **12** | Informed Consent | Did the patient give informed consent? Please provide if requested. | Declarations — Consent for Publication: written informed consent for clinical details and photographs obtained from patient's legal guardian. | **☑ Present** |
| **13a** | Discussion — Strengths & Limitations | Strengths and limitations of the case report | Discussion — Lessons Learned section + Limitations paragraph: strengths include high-quality operative photographs absent from prior literature; limitations include unavailability of original MRI images (referred with report only) and absence of intraoperative ultrasonography (not available in LMIC public surgical centre; manual testicular separation maneuver provided equivalent localisation). | **☑ Present** |
| **13b** | Discussion — Differential Diagnosis | Relevant medical literature referred to in this case | Clinical Reasoning and Diagnostic Challenges section: hydrocele [18,19], inguinal hernia [9], lipoblastoma [15–17], misdiagnosis rates [11], cognitive bias and diagnostic anchoring [13,14], prior pediatric scrotal lipoma cases [3,4,11,12] | **☑ Present** |
| **13c** | Rationale for Conclusions | Rationale for conclusions and key recommendations | Conclusions paragraph: expands anatomical spectrum; highlights limitations of clinical/imaging judgment in rare conditions; demonstrates feasibility of complete en bloc excision; 4 take-home clinical learning points enumerated in Lessons Learned section. | **☑ Present** |
